# Adrenergic control of the cardiovascular system in deer mice native to high altitude

**DOI:** 10.1016/j.crphys.2022.01.006

**Published:** 2022-01-29

**Authors:** Oliver H. Wearing, Derek Nelson, Catherine M. Ivy, Dane A. Crossley, Graham R. Scott

**Affiliations:** a Department of Biology, McMaster University, Hamilton, ON, Canada; b Department of Biological Sciences, University of North Texas, Denton, TX, USA

**Keywords:** Hypoxia, High-altitude adaptation, Autonomic control, Cardiovascular regulation, Cardiac function

## Abstract

Studies of animals native to high altitude can provide valuable insight into physiological mechanisms and evolution of performance in challenging environments. We investigated how mechanisms controlling cardiovascular function may have evolved in deer mice (*Peromyscus maniculatus*) native to high altitude. High-altitude deer mice and low-altitude white-footed mice (*P. leucopus*) were bred in captivity at sea level, and first-generation lab progeny were raised to adulthood and acclimated to normoxia or hypoxia. We then used pharmacological agents to examine the capacity for adrenergic receptor stimulation to modulate heart rate (*f*_H_) and mean arterial pressure (*P*_mean_) in anaesthetized mice, and used cardiac pressure-volume catheters to evaluate the contractility of the left ventricle. We found that highlanders had a consistently greater capacity to increase *f*_H_ via pharmacological stimulation of β_1_-adrenergic receptors than lowlanders. Also, whereas hypoxia acclimation reduced the capacity for increasing *P*_mean_ in response to α-adrenergic stimulation in lowlanders, highlanders exhibited no plasticity in this capacity. These differences in highlanders may help augment cardiac output during locomotion or cold stress, and may preserve their capacity for α-mediated vasoconstriction to more effectively redistribute blood flow to active tissues. Highlanders did not exhibit any differences in some measures of cardiac contractility (maximum pressure derivative, d*P*/dt_max_, or end-systolic elastance, E_es_), but ejection fraction was highest in highlanders after hypoxia acclimation. Overall, our results suggest that evolved changes in sensitivity to adrenergic stimulation of cardiovascular function may help deer mice cope with the cold and hypoxic conditions at high altitude.

## Highlights

• High-altitude deer mice have evolved increased aerobic capacity in hypoxia.
• Cardiovascular regulation was examined in normoxia and chronic hypoxia.
• Highland mice had increased capacity for β_1_-adrenergic stimulation of heart rate.
• Hypoxia reduced vascular α-adrenergic sensitivity in lowland but not highland mice.
• Cardiac ejection fraction was elevated in highland mice in chronic hypoxia.

## 1 Introduction

The mechanisms underlying the evolution of complex performance traits is a central and unresolved topic in evolutionary physiology (Dalziel et al., 2009; Garland et al., 2016; Garland and Carter, 1994; Scott and Dalziel, 2021). Studies of aerobic performance in endotherms that are native to high altitude can shed valuable insight into this topic (McClelland and Scott, 2019; Monge and Leon-Velarde, 1991; Storz, 2021; Storz et al., 2015; Wearing and Scott, 2021). High-altitude environments are cold and hypoxic, which challenges the ability of endotherms to maintain sufficient rates of O_2_ supply to meet the O_2_ demands of thermogenesis and locomotion. To help overcome this challenge, several vertebrate taxa that are native to high altitude – including some human populations – have evolved (genetically-based) increases in aerobic capacity (maximal O_2_ consumption, *V*O_2_max, during exercise or acute cold exposure) in hypoxia relative to their low-altitude counterparts (Brutsaert, 2016; Cheviron et al., 2013; Schippers et al., 2012). High-altitude environments can also lead to plastic increases in *V*O_2_max in response to exposure during adulthood (e.g., acclimatization) or early life (i.e., developmental plasticity) (Ivy et al., 2021; Storz and Cheviron, 2021; Storz and Scott, 2019; Tate et al., 2020). Therefore, both evolved changes and plasticity in the physiological determinants of oxygen transport (e.g. pulmonary, cardiovascular, etc.) appear to contribute to enhancing *V*O_2_max in high-altitude natives (Chen et al., 1997; Tate et al., 2020). However, the regulatory mechanisms that control these functional changes are not well understood in many high-altitude taxa.

Cardiac output and the preferential distribution of blood flow to metabolically active tissues are important determinants of aerobic capacity during exercise or thermogenesis, and adrenergic receptor stimulation is an important regulator of these processes. At the onset of exercise, sympathoadrenal activation (i.e., activation of the sympathetic nervous system and/or release of catecholamines from the adrenal medulla) leads to stimulation of cardiac output and relative redistribution of blood flow towards working muscles (Christensen and Galbo, 1983). Similarly, cold exposure leads to sympathoadrenal activation that increases cardiac output and redirects blood flow towards thermogenic muscles and brown adipose tissue (BAT) (Foster and Frydman, 1979; Landsberg et al., 1984). Therefore, sympathoadrenal activation and the resultant tissue responses to adrenergic stimulation are critical for supporting high metabolic rates. However, environmental hypoxia also leads to sympathoexcitation via the hypoxic chemoreflex, and chronic exposure to hypoxia (such as occurs at high altitude) can lead to persistent and prolonged sympathetic activation (Calbet, 2003; Hansen and Sander, 2003; Kuwahira et al., 1993a, 1993b; Richalet, 2016; Saito et al., 1988; Sander, 2016; Simpson et al., 2021; Storz and Scott, 2019). If left unabated, prolonged sympathetic activation due to chronic hypoxia is often associated with reductions in tissue sensitivity to adrenergic stimulation (Berthelsen et al., 2020; Fischetti et al., 2000; Ueno et al., 1997). However, this desensitization to adrenergic stimulation in response to chronic hypoxia could constrain or disrupt the cardiovascular responses to exercise or thermogenesis. This could have detrimental consequences in high-altitude natives, particularly in small endotherms that must sustain high rates of thermogenesis throughout the year to cope with cold temperatures (Hayes, 1989). Whether the sensitivity to adrenergic stimulation has evolved in high-altitude natives to overcome this issue and maintain appropriate cardiovascular responses to exercise and thermogenesis has yet to be resolved.

Previous studies have shown that evolved changes in autonomic control are idiosyncratic in humans native to high altitude. For example, while muscle sympathetic nerve activity (MSNA) measured at rest increases in lowland-native humans after 10–20 days at high altitude, MSNA is lower in highland-native Sherpa residing at high altitude (Simpson et al., 2019). In contrast, Andeans native to high altitude have resting MSNA resembling that of lowlanders visiting high altitude (Lundby et al., 2018). Therefore, there appears to be taxonomic differences in whether evolved mechanisms have arisen to reduce sympathetic activity in chronic hypoxia and thus help improve tissue blood flow and oxygen delivery at rest (Simpson et al., 2021). However, less is known about sympathetic activation during exercise, and whether the capacities for sympathetic responses have evolved in high-altitude humans. Furthermore, relatively little attention has been paid to adrenergic control in non-human animals native to high altitude. Although there is some evidence that some highland animals have reduced adrenergic sensitivity and/or receptor densities (León-Velarde et al., 1996; Pichon et al., 2013), these previous studies did not control for rearing environment and the results could have been explained by developmental hypoxia exposure.

Deer mice (*Peromyscus maniculatus*) native to high altitude are a powerful model for elucidating the cardiovascular mechanisms underpinning the evolution of aerobic performance. High-altitude populations sustain higher field metabolic rates than their low-altitude counterparts, likely to meet the increased oxygen demands of thermogenesis and the need to move greater distances to find food (Hayes, 1989). Increased thermogenic *V*O_2_max imparts a survival advantage and is likely under directional selection during harsh winters at high altitude (Hayes and O'Connor, 1999). As a result of selection, high-altitude deer mice have evolved increased thermogenic *V*O_2_max in hypoxia compared to low-altitude populations of deer mice and to white-footed mice, a congeneric species that is restricted to low altitudes (Chappell et al., 1988; Chappell and Snyder, 1984; Cheviron et al., 2012, 2013; Tate et al., 2017, 2020). Evolved changes across the oxygen transport pathway contribute to this increased *V*O_2_max, and high capacities for cardiac output and tissue O_2_ extraction at *V*O_2_max appear to play particularly important roles (Lui et al., 2015; Mahalingam et al., 2017; Natarajan et al., 2015; Scott et al., 2015; Snyder et al., 1982; Storz et al., 2007, 2010, 2019; Tate et al., 2017, 2020; Wearing et al., 2021; West et al., 2021). These differences in cardiac output and tissue O_2_ extraction could result from changes in adrenergic regulation of the heart and vasculature and/or contractile function of the heart, but these possibilities have yet to be examined.

In this study, we hypothesized that high-altitude deer mice have evolved changes in adrenergic control of cardiovascular function and changes in cardiac contractility to augment cardiac output and blood flow to thermogenic tissues in hypoxia. We predicted that adrenergic control of cardiovascular function would be altered in high-altitude mice in two ways, specifically: 1) β_1_-adrenergic receptor stimulation would result in greater increases in heart rate in highland mice than in lowland mice; and 2) changes in α-adrenergic receptor sensitivity that occur in lowland mice in chronic hypoxia would be attenuated in highland mice. We also predicted that highland deer mice would have hearts with greater contractility – assessed from pressure-volume (P–V) relationships of the left ventricle – facilitating greater stroke volumes at *V*O_2_max compared to lowland mice.

## 2 Materials and methods

### 2.1 Animals and environmental exposures

Lab-raised breeding colonies were derived from wild adult *Peromyscus* mice caught at high and low altitudes. Deer mice from a high-altitude population (*P. m. rufinus*) were caught around the summit of Mount Evans at 4350 m above sea level (Clear Creed County, CO, USA at 39°35′18”N, 105°38′38”W), and low-altitude white-footed mice (*P. leucopus*) were caught in the Great Plains of Nebraska at 430 m above sea level (Nine Mile Prairie, Lancaster County, NE, USA at 40°52′12”N, 96°48′20.3”W). Following transportation to McMaster University (near sea level), mice were bred to produce first-generation (G_1_) lab progeny. These G_1_ progeny of highland deer mice and lowland white-footed mice were raised to 6 months of age in common normoxic conditions before use in experiments. All mice were held at standard lab temperature (24–25 °C) and photoperiod (12 h light: 12 h dark) with unlimited access to standard rodent chow and water. All animal protocols followed guidelines established by the Canadian Council on Animal Care and were approved by the McMaster University Animal Research Ethics Board.

Starting at approximately 6 months of age, G_1_ mice were exposed for 6–8 weeks to one of two environmental conditions: (i) normobaric normoxia (∼21 kPa O_2_) in ambient air, or (ii) hypobaric hypoxia (12 kPa O_2_, equivalent to hypoxia at ∼4300 m above sea level). Hypoxia was achieved and maintained using hypobaric chambers as described previously (Lui et al., 2015; McClelland et al., 1998). Cages were cleaned twice per week, which required that hypoxic mice experience brief (<20 min) periods of normoxia. After the 6–8 week exposure period, mice were then used for measurements of the capacity for adrenergic control of the cardiovascular system (Section 2.2) or cardiac contractility (Section 2.3). The total number of individuals used in each treatment group in this study were as follows: 10 highland deer mice in normoxia (4 females, 6 males); 9 highland deer mice in hypoxia (3 females, 6 males); 11 lowland white-footed mice in normoxia (6 females, 5 males); 12 lowland white-footed mice in hypoxia (5 females, 7 males).

### 2.2 Adrenergic control of the cardiovascular system

We determined the capacity for adrenergic receptors to modulate cardiovascular function by measuring the difference between maximal stimulation with selective agonists and maximal inhibition with selective antagonists in isoflurane anaesthetized mice. Receptor-specific adrenergic agonists and antagonists were prepared fresh daily. The compounds were dissolved in sterile physiological saline (0.9% NaCl in deionized water) and prepared for intravenous (IV) injection (receptor agonists, 0.0033 ml per g body mass) or intraperitoneal (IP) injection (receptor antagonists, 0.02 ml per g body mass). Dobutamine hydrochloride (2 mg kg^−1^ IV; Cayman Chemical, Ann Arbor, MI, USA) and metoprolol tartrate (10 mg kg^−1^ IP) were used to stimulate and block the positive chronotropic action of cardiac β_1_-adrenegic receptors, respectively. Phenylephrine hydrochloride (0.2 mg kg^−1^ IV) and phentolamine hydrochloride (15 mg kg^−1^ IP) were used to stimulate and block the vasoconstrictive action of vascular α-adrenergic receptors, respectively. Preliminary experiments confirmed that the doses used elicited maximal effects on heart rate (*f*_H_) or mean arterial pressure (*P*_mean_). All pharmacological compounds were purchased from Sigma-Aldrich Canada (Oakville, ON, Canada) unless stated otherwise.

Each mouse first underwent surgical catheterization of the right jugular vein and left carotid artery. The mouse was first placed in an anaesthetic induction chamber and anaesthesia was induced using 3% isoflurane balanced with O_2_ at a flow rate of 1500 ml min^−1^. The mouse was then placed supine on a heating pad, and a nose cone was used to administer 1.5% isoflurane in the inspired gas to maintain a stable surgical plane of anaesthesia. This dose of isoflurane (1.5%) is below the concentration at which effects of anaesthesia on heart rate, blood pressure, myocardial contractility, and left ventricular diastolic function have been observed in rats (3% isoflurane) (Yang et al., 2014). The mouse was then instrumented with a rectal thermocouple (RET-3-ISO, Physitemp) and the heating pad was regulated to maintain a core body temperature of 35–40 °C throughout the procedure. The ventral skin of the neck was shaved and wiped clean using an isopropyl alcohol swab, and a 15-mm incision was made along the midline of the neck. The salivary glands were gently pushed aside by blunt dissection to reveal the trachea and the carotid artery. The artery was carefully isolated from the surrounding tissue (including the vagus nerve) at a location proximal to the carotid bifurcation, and was conclusively cannulated with a microrenathane catheter (MRE025, Braintree Scientific, Braintree, MA, USA) filled with 100 units ml^−1^ heparin dissolved in 0.9% sterile saline. The catheter was advanced approximately 15mm so the tip was in the aortic arch, the catheter was secured to the carotid artery with suture, and the vessel was ligated distal to the incision used to insert the catheter. The other end of this catheter was connected to a fluid-filled pressure transducer (model MLT0699, ADInstruments) that allowed for acquisition of arterial pressure data (200 Hz) using a PowerLab 8/35 and LabChart 8 Pro software (ADInstruments). From this pressure trace, we recorded *P*_mean_ and *f*_H_. A second catheter filled with 0.9% saline (without heparin) was similarly inserted approximately 15 mm into the right jugular vein and the other end was connected to a 1-ml syringe with a blunted 27G needle.

Measurements were taken after catheterization once cardiovascular parameters had stabilized. The effects of manipulating α- and β_1_-adrenergic receptor stimulation were assessed in a subset of mice in each experimental group. Baseline measurements of *P*_mean_ and *f*_H_ were recorded for 5 min to ensure cardiovascular function was stable. Venous blood was withdrawn into the venous catheter until blood reached the needle hub, and the catheter was clamped using haemostats. A syringe containing phenylephrine was then connected to the needle hub and the drug dose was injected. The response was then recorded and the maximum *P*_mean_ achieved over a 1-s recording period was designated as the drug response (typically occurring within 5 s post-injection). Once *P*_mean_ and *f*_H_ had returned to baseline levels, phentolamine was injected IP, and the minimum *P*_mean_ over 1 s was recorded (typically occurring 10–15 min post-injection). Following this, a similar protocol was carried out using IV dobutamine followed by IP metoprolol to measure maximum and minimum *f*_H_. A separate subset of mice were used to examine the effects of β_1_-adrenergic receptor stimulation alone using the same procedure. Samples sizes for all mice that underwent β_1_-adrenergic receptor stimulation were as follows: highland deer mice in normoxia, n = 7 (3 females, 4 males); highland deer mice in hypoxia, n = 6 (2 females, 4 males); lowland white-footed mice in normoxia, n = 8 (5 females, 3 males); lowland white-footed mice in hypoxia, n = 8 (3 females, 5 males). Samples sizes for the subset of mice used for α-adrenergic receptor stimulation were as follows: highland deer mice in normoxia, n = 4 (2 females, 2 males); highland deer mice in hypoxia, n = 4 (1 female, 3 males); lowland white-footed mice in normoxia, n = 4 (3 females, 1 male); lowland white-footed mice in hypoxia, n = 3 (2 females, 1 male). Due to the potential effects of anaesthesia on metabolism and the tone of the autonomic nervous system (Skovsted and Sapthavichaikul, 1977), which could influence baseline cardiovascular values, we focussed on the difference between maximal and minimal cardiovascular values elicited pharmacologically to quantify the ability for the targeted adrenergic receptors to regulate cardiovascular function. Upon completion of the protocol, animals were euthanized under anaesthesia by cervical dislocation, and hearts were excised to determine ventricle masses.

### 2.3 Contractility of the heart

**Fig. 1:**
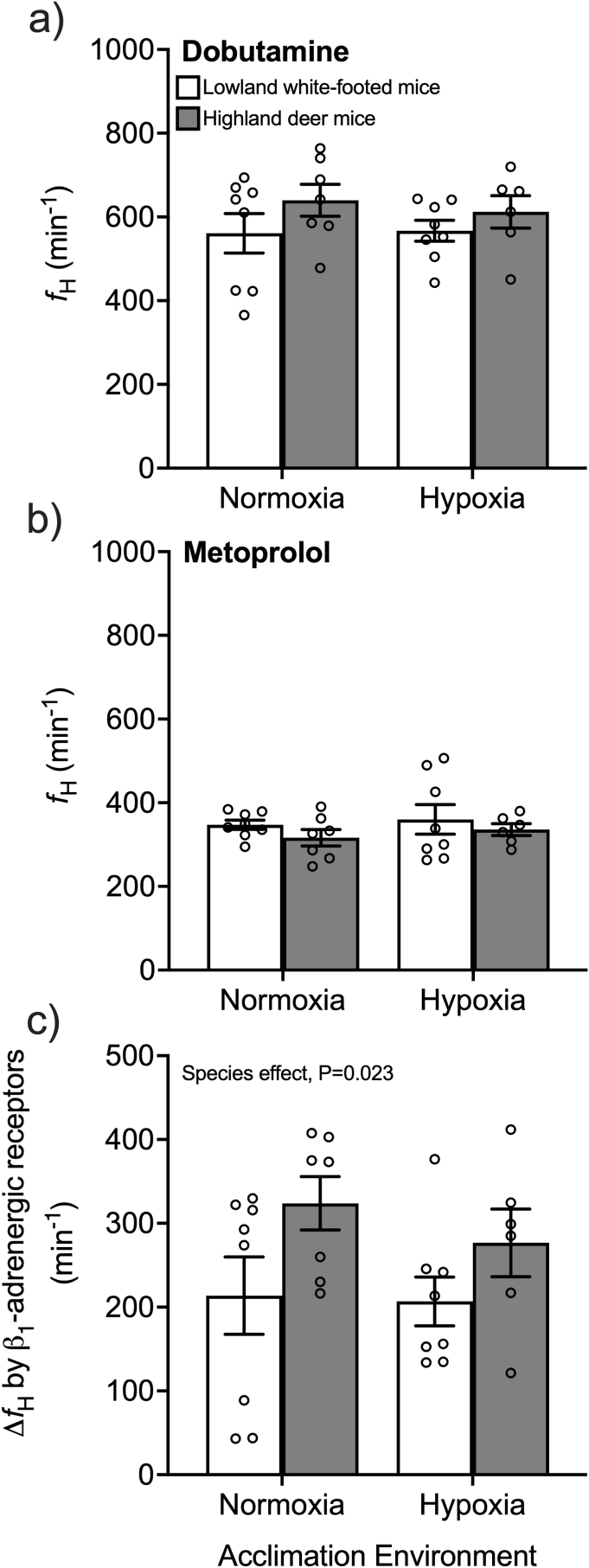
Heart rate (*f*_H_) after pharmacological stimulation of cardiac β_1_-adrenergic receptors by dobutamine (a) followed by blockade with metoprolol (b) in white-footed mice (a species restricted to low altitude) and in deer mice from a population native to high altitude. Mice were acclimated to normoxia (21 kPa O_2_) or hypobaric hypoxia (12 kPa O_2_) for 6–8 weeks. c) Change in heart rate (Δ*f*_H_) by stimulation of β_1_-adrenergic receptors was calculated as the difference between maximal stimulation with dobutamine and blockade with metoprolol. Bars display mean ± SEM with individual data as circles. *P < 0.05 between species within an acclimation environment. ^†^P < 0.05 between acclimation environments within a species.

The contractile function of the left ventricle was measured using pressure-volume catheters in each experimental group. Each mouse was first anaesthetized with isoflurane in an anaesthetic induction chamber, moved to a heating pad where body temperature was maintained and they breathed through a nose cone, and then maintained at a surgical plane of anaesthesia with 1.5% isoflurane as described above. The ventral skin of the neck, thorax and abdomen was then shaved and cleaned, and a 50-mm incision was made along the body midline from the chin. The salivary glands and underlying muscles in the neck were gently pushed aside by blunt dissection to reveal the trachea. A small slit was made between the larynx and the first tracheal ring, and a mouse endotracheal tube was quickly inserted into the trachea, secured with suture, connected to a ventilator (VentElite Small Animal Ventilator, Harvard Apparatus, Holliston, MA, USA), and used to initiate artificial ventilation with air (300-μl tidal volume at 130 breaths per min; based on previous ventilation measurements by Ivy and Scott, 2017). Isoflurane (1.5%) in the ventilated air was used to maintained a surgical plane of anaesthesia. A laparotomy was then performed by making an approximately 3-cm midline incision through the skin and abdominal wall from the xyphoid process. The liver was carefully retracted caudad to expose the diaphragm, which was then punctured to make an approx. 1-cm medial incision at the midline to expose the apex of the heart. After carefully peeling back the pericardium from the apex, the left ventricle was punctured quickly and carefully at the apex using a 21G needle, and a pressure-volume catheter (1.2-F diameter pressure-volume catheter FTH-1212B-3518, Transonic Scisense, London, ON, Canada) was advanced as quickly as possible into the puncture hole. This catheter was connected to a Scisense ADVantage 5.0 control unit (Transonic Scisense) that interfaced with a PowerLab 8/35 (ADInstruments, Colorado Springs, CO, USA), and data was visualised in real-time and recorded using LabChart 8 Pro software (ADInstruments). Once proper catheter placement was confirmed by the production of stereotypical ventricular pressure-volume (P–V) loops (see Fig. 1), the preparation was allowed to stabilize before left ventricle parameters were recorded, which occurred within 5 min of catheter placement. Loops were visually inspected to choose representative loops for each animal without catheter placement artifacts, which were then used for parameter calculation using the automated LabChart 8 Pro P–V Loop module (see below). We were thus able to obtain P–V loops for each group with the following sample sizes: highland deer mice in normoxia, n = 3 (1 female, 2 males); highland deer mice in hypoxia, n = 3 (1 female, 2 males); lowland white-footed mice in normoxia, n = 3 (1 female, 2 males); lowland white-footed mice in hypoxia, n = 4 (2 females, 2 males). However, given the challenges of this technique, there were additional individuals in each group for which we could not obtain P–V loops and no data are reported. Mice were then euthanized by cervical dislocation, and hearts were excised to determine ventricle masses.

Left ventricle (LV) parameters were calculated using the automated LabChart 8 Pro P–V Loop module. Pressure and volume traces were simultaneously acquired using the P–V catheter, and used to produce P–V loops. The individual LV pressure trace was used to obtain maximal (*P*_max_) and minimal (*P*_min_) LV pressures per heart beat, as well as *P*_mean_ calculated as $\frac{P_{max}+2P_{min}}{3}$, and developed pressure, *P*_dev_ ($P_{max}-P_{min}$). This pressure trace was also used to calculate *f*_H_. Stroke volume was calculated as the difference between the minimum and maximum (*V*_max_) LV volumes per beat, and was multiplied by *f*_H_ to calculate cardiac output. Stroke work was then calculated as the product of *P*_mean_ and stroke volume. Finally, we acquired three indices of cardiac contractility: d*P*/dt_max_, the maximal rate of LV pressure increase per beat acquired from the pressure trace; ejection fraction (EF, %), which was calculated as $\frac{Stroke\;volume}{V_{max}}\times 100$ per beat; and the slope of the end-systolic pressure-volume relationship, the end-systolic elastance (E_es_), which is calculated as end-systolic pressure divided by end-systolic volume.

### 2.4 Statistical analysis

We used linear mixed effects models to test for the effects of species, chronic hypoxia exposure, and their interaction using the lme4 package (Bates et al., 2015) in R Studio (Version April 1, 1103, RStudio Public Benefit Corporation, Boston, MA, USA). Initial models were run including effects of body mass, individual (for repeated-measures drug injections), family, and sex as random factors as appropriate. If body mass, family or sex had P values above 0.1 in the initial model, they were removed by stepwise backward deletion (starting with the term with the highest P value) and the model was re-run until all terms in the model (with the exception of fixed factors and individual subject) had P values below 0.1. The full results of final statistical models are included in the supplementary material (Tables S1–S4, see Supplementary Material) and the salient findings are reported in the Results. Family was included in only 4 of the models, and sex was included in only one model. Tukey's HSD post hoc tests were performed to test for pairwise differences between species within an acclimation or injection group, between acclimations within a species or injection group, and between injections within a species or acclimation group. Data are presented as individual values (small circles) and mean ± SEM (bars) unless otherwise stated.

## 3 Results

### 3.1 Highland deer mice had similar sized hearts but relatively small right ventricles compared to white-footed mice

We measured body mass and ventricle masses across all the highland deer mice and lowland white-footed mice used in this study. Highland deer mice were approximately 30% smaller than white-footed mice (main effect of species on body mass, P = 0.002) (Table 1). This difference was expected based on the known difference in body size between species, and it is not unique to the high-altitude population (within deer mice, low- and high-altitude populations have similar body masses) (Tate et al., 2020). When the effects of variation in body mass was accounted for as a covariate in statistical models, there were no overall differences between species or acclimation environments on total ventricle mass (species effect, P = 0.951; acclimation effect, P = 0.458), left ventricle plus septum (LV + S) mass (species effect, P = 0.481; acclimation effect, P = 0.173), or right ventricle (RV) mass (species effect, P = 0.088; acclimation effect, P = 0.150). Although the species differences were not significant, highlanders tended to have larger total ventricle and LV + S masses and smaller RV mass when expressed relative to body mass (Table 1). As a result, the ventricular mass ratio also known as Fulton's index, RV/(LV + S), was ∼22–27% lower in highlanders compared to lowlanders (species effect, P < 0.001). This difference offset the increase in RV/(LV + S) after hypoxia acclimation (acclimation effect, P = 0.016), such that RV/(LV + S) was lower on average in hypoxic highlanders than in normoxic lowlanders (Table 1).

**Table 1:** Body and heart masses in white-footed mice (‘lowlander’, *Peromyscus leucopus*) and in deer mice from a population native to high altitude (‘highlander’, *P. maniculatus*), each of which were acclimated to normoxia (21 kPa O_2_) or hypobaric hypoxia (12 kPa O_2_) for 6–8 weeks.

	Normoxia		Hypoxia	
	Lowlander (n = 11)	Highlander (n = 10)	Lowlander (n = 12)	Highlander (n = 9)
Body mass, g	28.9 ± 2.2	20.7 ± 1.1*	30.1 ± 1.2	20.6 ± 1.1*
Total ventricle mass, mg g^−1^	4.21 ± 0.21	4.78 ± 0.15	3.98 ± 0.20	4.61 ± 0.15
RV mass, mg g^−1^	0.721 ± 0.026	0.631 ± 0.034	0.806 ± 0.087	0.725 ± 0.035
LV + S mass, mg g^−1^	3.48 ± 0.19	4.15 ± 0.14	3.17 ± 0.15	3.89 ± 0.14
RV/(LV + S)	0.210 ± 0.011	0.153 ± 0.008*	0.255 ± 0.025	0.188 ± 0.010*^†^

*Significant species effect within acclimation environment, P < 0.05. ^†^Significant acclimation effect within a species. RV, right ventricle; LV + S, left ventricle and septum; RV/LV + S, right ventricle to left ventricle and septum ratio. Ventricle masses were obtained from a subset of mice within each group (n = 7).

### 3.2 Highland deer mice have greater scope for adrenergic stimulation of the cardiovascular system

We used pharmacological agents to maximally stimulate and block β_1_-adrenergic receptors to determine their ability to increase heart rate (*f*_H_) in anaesthetized mice. There were no significant differences between species nor significant effects of hypoxia acclimation on *f*_H_ before pharmacological manipulation (baseline *f*_H_ before injections: normoxic lowlander, 516 ± 39 min^−1^; normoxic highlander, 540 ± 14 min^−1^; hypoxic lowlander, 509 ± 21 min^−1^; hypoxic highlander, 477 ± 21 min^−1^). As expected, β_1_-adrenergic receptor drugs had a significant effect on *f*_H_ (P < 0.001), with *f*_H_ being 206 min^−1^ to 324 min^−1^ higher after dosing with the β_1_-adrenergic receptor agonist dobutamine (Fig. 1a) compared to subsequent dosing with the receptor antagonist metoprolol (Fig. 1b). We then calculated the change in *f*_H_ between the β_1_-adrenergic receptor agonist and antagonist (*i.e.* Δ*f*_H_) as an indication of the potential scope for adrenergic stimulation of *f*_H_ (Fig. 1c). Highland deer mice had 34–51% higher Δ*f*_H_ on average than lowland white-footed mice (species effect, P = 0.023).

**Fig. 2:**
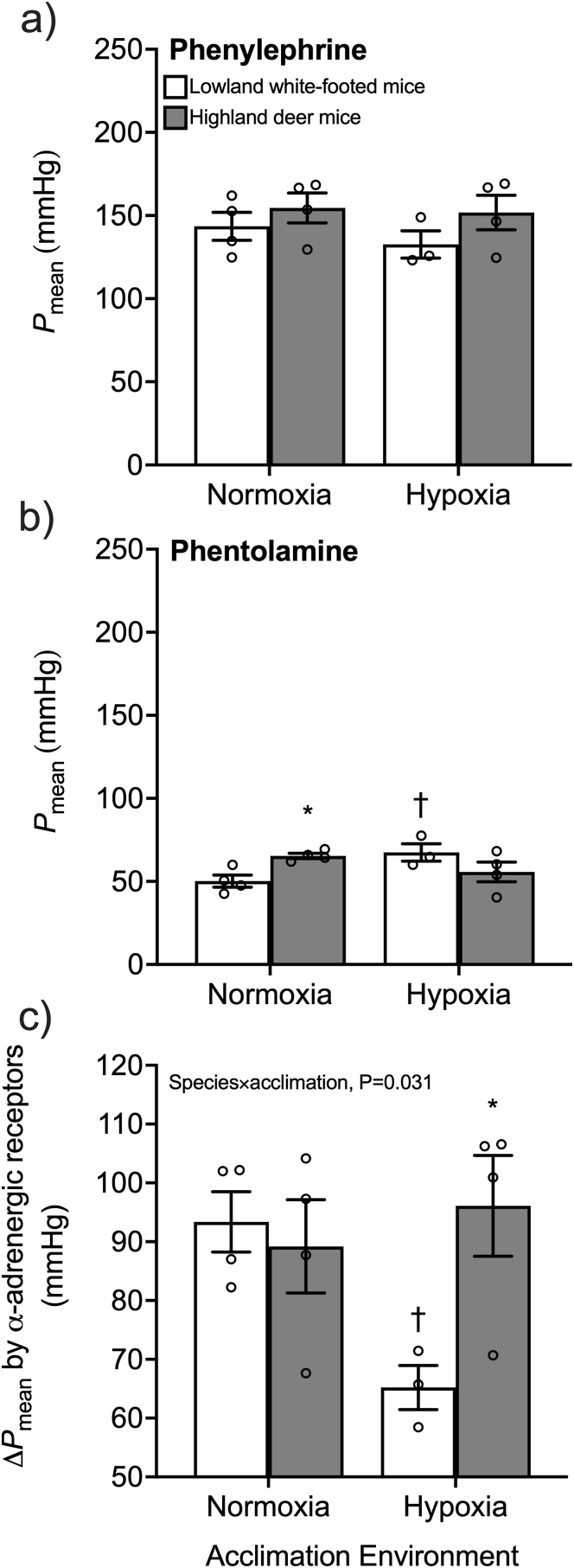
Mean arterial pressure (*P*_mean_) after pharmacological stimulation of vascular α-adrenergic receptors by phenylephrine (a) followed by blockade with phentolamine (b) in white-footed mice (a species restricted to low altitude) and in deer mice from a population native to high altitude. Mice were acclimated to normoxia (21 kPa O_2_) or hypobaric hypoxia (12 kPa O_2_) for 6–8 weeks. c) Change in mean arterial pressure (Δ*P*_mean_) caused by stimulation of α-adrenergic receptors was calculated as the difference between maximal stimulation with phenylephrine and blockade with phentolamine. Bars display mean ± SEM with individual data as circles. *P < 0.05 between species within an acclimation environment. ^†^P < 0.05 between acclimation environments within a species.

We pharmacologically stimulated and blocked α-adrenergic receptors to determine the ability of these receptors to regulate blood pressure. There were no differences between species nor effects of hypoxia acclimation on mean arterial pressure (*P*_mean_) before pharmacological manipulation (baseline *P*_mean_: normoxic lowlander, 85.2 ± 8.3 mmHg; normoxic highlander, 91.9 ± 3.1 mmHg; hypoxic lowlander, 101.0 ± 4.6 mmHg; hypoxic highlander, 102.6 ± 9.4 mmHg). As expected, the α-adrenergic receptor drugs had a significant effect on *P*_mean_ (P < 0.001), with *P*_mean_ being 65–96 mmHg higher after dosing with the α-adrenergic receptor agonist phenylephrine (Fig. 2a) compared to subsequent dosing with the receptor antagonist phentolamine (Fig. 2b). Similar to the approach used for β_1_-receptor manipulation, we calculated the change in *P*_mean_ between the α-adrenergic receptor agonist and antagonist (*i.e.* Δ*P*_mean_) as an indication of the potential scope for adrenergic regulation of blood pressure (Fig. 2c). There was a significant 30% reduction in Δ*P*_mean_ after hypoxia acclimation in lowlanders, but no change in Δ*P*_mean_ in highlanders (species × environment interaction, P = 0.031). The former was associated with an increase in *P*_mean_ after phentolamine injection in lowlanders after hypoxia acclimation (Fig. 2b). We did not observe any significant effects of sex on α-adrenergic responses (nor on β_1_-adrenergic responses; Table S3), despite the potential for sex differences in adrenergic control of the cardiovascular system (Joyner et al., 2015; Vizgirda et al., 2002), but we likely lacked sufficient sample sizes of males and females to detect such differences.

### 3.3 Pressure-volume relationships and contractility of the left ventricle

**Fig. 3:**
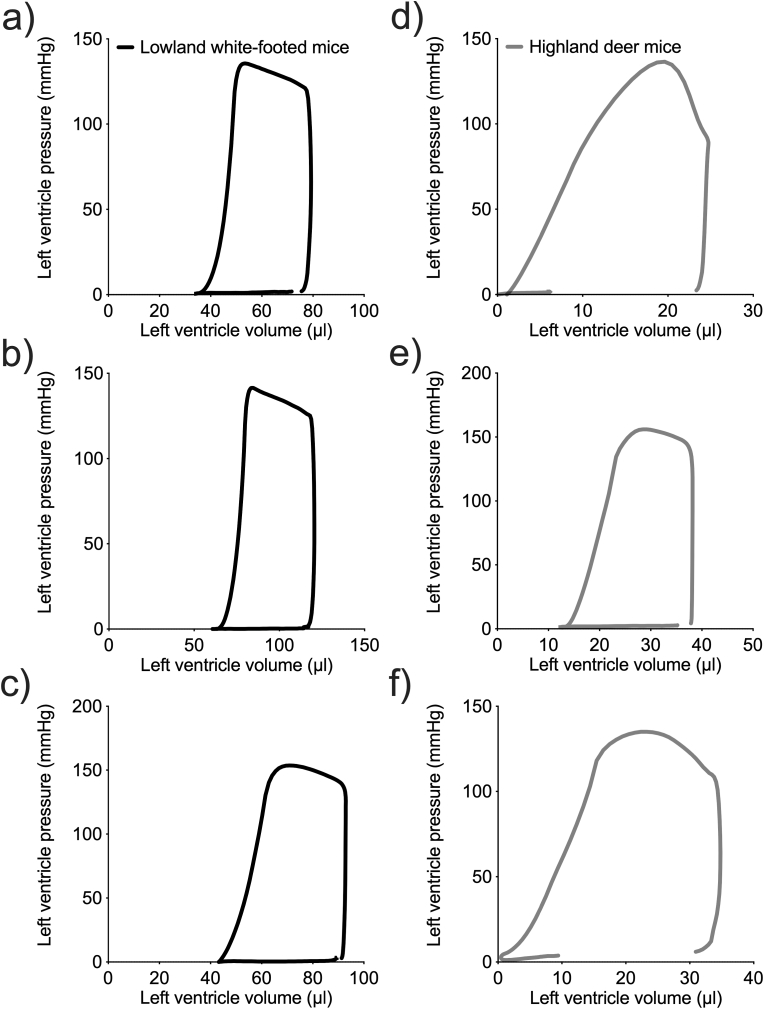
Representative pressure-volume (P–V) loops for the left ventricle of individual white-footed mice (a species restricted to low altitude) (a–c) and individual deer mice from a population native to high altitude (d–f) after hypoxia acclimation.

We measured pressure-volume relationships inside the left ventricle in anaesthetized mice. Pressure-volume (P–V) loops exhibited characteristic low-pressure filling phase (bottom), steep isovolumetric contraction phase (right), rising pressure during ejection phase (top), and isovolumetric relaxation phase (left) (Fig. 3). After accounting for effects of species differences in body mass as a covariate in statistical models, resting stroke volume, cardiac output, stroke work, and maximum and minimum ventricle volumes were lower overall in highlanders than in lowlanders (species effects, P ≤ 0.05). The differences in stroke volume, cardiac output, and stroke work were driven primarily by smaller values in highlanders in normoxia, but the species differences were no longer significant after hypoxia acclimation (Table 2). Otherwise, hypoxia acclimation significantly increased stroke work (acclimation effect, P = 0.023) and the pressure developed by contraction (*P*_dev_; acclimation effect, P = 0.046), and it also reduced minimum pressure (*P*_min_; acclimation effect, P = 0.041). There was a reduction in resting *f*_H_ in lowlanders after hypoxia acclimation (Table 2), but values were still within the ranges measured during the pharmacology manipulations (Fig. 1).

**Table 2:** Left ventricle parameters measured using intraventricular pressure-volume catheter in white-footed mice (‘lowlander’, *Peromyscus leucopus*) and in deer mice from a population native to high altitude (‘highlander’, *P. maniculatus*), each of which were acclimated to normoxia (21 kPa O_2_) or hypobaric hypoxia (12 kPa O_2_) for 6–8 weeks.

	Normoxia		Hypoxia	
	Lowlander (n = 3)	Highlander (n = 3)	Lowlander (n = 4)	Highlander (n = 3)
*f*_H_, min^−1^	577 ± 42	543 ± 66	405 ± 39^†^	530 ± 29
Stroke volume, μl g^−1^	1.094 ± 0.139	0.582 ± 0.077*	1.221 ± 0.028	1.025 ± 0.209
Cardiac output, ml min^−1^ g^−1^	0.631 ± 0.090	0.308 ± 0.033*	0.492 ± 0.037	0.544 ± 0.120
Stroke work, mmHg μl g^−1^	135.2 ± 15.3	61.2 ± 15.4*	165.4 ± 10.3	136.9 ± 25.1
*V*_max_, μl g^−1^	3.145 ± 0.282	1.770 ± 0.317*	3.129 ± 0.159	1.733 ± 0.310
*V*_min_, μl g^−1^	1.552 ± 0.243	0.783 ± 0.345	1.321 ± 0.204	0.225 ± 0.211
*P*_max_, mmHg	134 ± 4	113 ± 15	139 ± 6	143 ± 7
*P*_min_, mmHg	2.808 ± 1.774	3.729 ± 1.778	0.327 ± 0.479	0.166 ± 0.888
*P*_mean_, mmHg	49.7 ± 1.7	39.2 ± 6.6	41.3 ± 4.0	43.5 ± 4.6
*P*_dev_, mmHg	131 ± 2	110 ± 14	138 ± 7	142 ± 6
EF, %	34.7 ± 1.8	37.8 ± 11.3	39.4 ± 1.7	60.2 ± 6.7
d*P*/dt_max_, mmHg s^−1^	9314 ± 357	10,394 ± 2378	11,169 ± 825	12,974 ± 2682
E_es_, mmHg μl^−1^	1.93 ± 0.29	6.73 ± 2.11	3.30 ± 0.44	5.40 ± 2.75

*Significant species effect within acclimation environment, P < 0.05. ^†^Significant acclimation effect within a species, P < 0.05. *f*_H_, heart rate; *V*_max_, maximum left ventricle volume; *V*_min_, minimum left ventricle volume; *P*_max_, maximum left ventricle pressure; *P*_min_, minimum left ventricle pressure; *P*_mean_, mean left ventricle pressure; *P*_dev_, pressure developed by left ventricle contraction; EF, ejection fraction; d*P*/dt_max_, maximum derivative of pressure; E_es_, end-systolic elastance, which is the slope of the end-systolic pressure-volume relationship.

We used the aforementioned pressure-volume relationships to obtain load-dependent (maximum pressure derivative, d*P*/dt_max_, and ejection fraction, EF) and load-independent (end-systolic elastance, E_es_) indices of left ventricle contractility (Table 2). Neither d*P*/dt_max_ nor E_es_ differed between species (species effects, P = 0.466 and 0.067, respectively) or acclimation environments (acclimation effects, P = 0.235 and 0.947, respectively). In contrast, whereas ejection fraction was ∼35–40% in lowlanders, it rose to 60% in highlanders after hypoxia acclimation (acclimation effect, P < 0.001; species × environment interaction, P = 0.004) (Table 2, Table S4). The latter appeared to result from a lower minimum ventricle volume after hypoxia acclimation in highlanders (Table 2).

## 4 Discussion

High-altitude deer mice have evolved a suite of physiological changes across the oxygen transport pathway that aid in supplying oxygen to active tissues in an oxygen-limited environment, but the importance of changes in adrenergic control of cardiovascular function was previously unknown. We found that highland deer mice had a greater capacity than lowlanders to elevate heart rate via stimulation of β_1_-adrenergic receptors. Furthermore, whereas chronic hypoxia reduced the capacity for increasing blood pressure in response to α-adrenergic receptor stimulation in lowlanders, this capacity was preserved in chronic hypoxia in highlanders. These differences may help augment cardiac output and preserve the capacity for α-mediated vasoconstriction, to more effectively redistribute blood flow and improve O_2_ delivery to active tissues during locomotion or cold stress. High-altitude adaptation does not appear to have caused any substantial changes in the load-independent contractile function of the left ventricle, although highland deer mice exhibited high ejection fraction in chronic hypoxia. Overall, our results suggest that evolved changes in adrenergic regulation of the cardiovascular system may help highland deer mice cope with the cold and hypoxic conditions at high altitude.

### 4.1 Chronic hypoxia reduces vascular responses to adrenergic activation in low-altitude mice

The reduced capacity for responding to α-adrenergic stimulation after hypoxia acclimation in lowland white-footed mice could reflect a plastic vascular response resulting from chronic activation of the hypoxic chemoreflex. The hypoxic chemoreflex, initiated when the carotid bodies detect low arterial O_2_ levels, leads to sympathoadrenal activation, and the resulting α-mediated vasoconstriction can increase vascular resistance, restrict blood flow to some tissues, and induce systemic hypertension (Calbet, 2003; Hainsworth and Drinkhill, 2007; Hainsworth et al., 2007; Richalet, 2016; Rimoldi et al., 2016; Sander, 2016). The observed response of lowland white-footed mice to chronic hypoxia should help attenuate these effects by reducing the responsiveness of the systemic vasculature to catecholamines. This response could be explained by a downregulation of α-adrenergic receptor density, as previously described following hypoxia acclimation in other species (Fischetti et al., 2000; Ueno et al., 1997). However, these changes could come at the expense of other homeostatic processes that rely upon autonomic control of the vasculature, such as the baroreflex and the controlled re-distribution of blood flow to active tissues during locomotion and/or thermogenesis (e.g., skeletal muscles, brown adipose tissue).

Lowland white-footed mice demonstrated no plasticity in the capacity for a heart rate response to β_1_-adrenergic receptor stimulation, which differs from the expectation from previous studies in some other animals. Chronic hypoxia has been shown to reduce β-receptor sensitivity and density in cardiac tissue in some other species, and has been associated with reductions in maximal heart rate (Favret and Richalet, 2007; Kacimi et al., 1992; Kanai et al., 2001; León-Velarde et al., 1996; Voelkel et al., 1981). Our observations suggesting that this does not occur may explain why heart rates measured at VO_2_max (maximal rate of O_2_ consumption during thermogenesis) are not reduced after hypoxia acclimation in *Peromyscus* mice (Tate et al., 2017, 2020). Our results are therefore supportive of the growing appreciation that interspecific differences between low-altitude mammals (e.g., old world mice versus rats) can alter the responses to and tolerance of chronic hypoxia (Arias-Reyes et al., 2021; Jochmans-Lemoine et al., 2015).

### 4.2 High-altitude deer mice have increased capacity for adrenergic control of the cardiovascular system in chronic hypoxia

Our finding that highlanders have an enhanced capacity to increase heart rate in response to β_1_-adrenergic stimulation provides a potential mechanism for evolved increases in thermogenic capacity and maximal cardiac output in hypoxia. High-altitude deer mice have evolved greater thermogenic capacity than both low-altitude conspecifics and low-altitude white-footed mice, likely as a result of strong directional selection in cold alpine environments (Chappell et al., 1988; Chappell and Snyder, 1984; Cheviron et al., 2012; Cheviron et al., 2013; Hayes and O'Connor, 1999). We have previously demonstrated that this increase in aerobic capacity is associated with evolved changes across the oxygen transport pathway (Lui et al., 2015; Ivy et al., 2020; Mahalingam et al., 2017; Wearing et al., 2021; West et al., 2021a,b), including higher maximal heart rate and/or stroke volume at VO_2_max in chronic hypoxia (Tate et al., 2017, 2020). Our results here suggest that the former may be achieved at least in part from a greater response to stimulation of cardiac β_1_-adrenergic receptors upon activation of the sympathetic nervous system during cold exposure (Morrison et al., 2008; Richalet, 2016; Sander, 2016; Storz and Scott, 2019). These findings are in stark contrast to high-altitude pikas and humans, which exhibit lower β_1_-adrenergic sensitivity or tone compared to lowlanders, and in the former case this was associated with lower receptor mRNA expression (Pichon et al., 2013; Zhuang et al., 1993). However, such differences may not be surprising in light of previous findings that highland deer mice maintain very high metabolic rates in the wild (Hayes, 1989), in contrast to high-altitude pikas that instead suppress metabolism to cope at high altitude (Speakman et al., 2021). The benefit of an enhanced capacity for cardiac β_1_-adrenergic stimulation may be restricted to high-altitude taxa that have high metabolic demands for thermogenesis or locomotion at high altitude.

The lack of any effects of chronic hypoxia on α-adrenergic responses in highlanders may help maintain autonomic vascular control at normal levels. This could be advantageous for preserving the baroreflex and the ability to re-distribute blood flow in response to metabolic need, but it could potentiate systemic hypertension and blood flow restriction if α-receptors are chronically stimulated by the hypoxic chemoreflex. However, we recently found that high-altitude deer mice have lower circulating epinephrine levels than low-altitude mice, due to an evolved reduction in catecholamine secretion from the adrenal medulla (Scott et al., 2019). As such, chronic hypoxia may not lead to chronic adrenergic stimulation via humoral means in highlanders, negating the need to downregulate vascular α-receptors to avoid hypertension and blood flow restriction. It is possible that highland mice have also evolved increased vasodilatory tone on the vasculature, as observed in highland-native humans from Tibet that show elevated circulating levels of nitric oxide products (Erzurum et al., 2007), but this possibility remains unexamined.

We observed equivocal evidence that the left ventricle of highland deer mice has greater contractility than lowland mice, based on measurements of pressure-volume relationships in anaesthetized mice. On the one hand, there were no species differences (or effects of hypoxia acclimation) on the maximum pressure derivative (d*P*/dt_max_) or end-systolic elastance (E_es_; an index of load-independent contractility). On the other hand, EF was highest in highland mice after hypoxia acclimation. However, given the discordance between these measures of contractility, and the necessity that measurements were made on a relatively small sample size of anaesthetized mice, it remains unclear if highland mice have greater left ventricle contractility at the much higher cardiac outputs and stroke volumes at *V*O_2_max. Variation in metabolism, systemic vascular resistance, and venous return under anaesthesia could explain some of the observed variation in left ventricle volumes and pressures, all of which would thus be expected to change at the high metabolic rates during intense aerobic thermogenesis. Therefore, although our results suggest there are few differences in cardiac contractility in highland deer mice, future work is needed to determine if this is also the case at higher metabolic rates or if increases in contractility help facilitate increases in stroke volume and cardiac output to augment *V*O_2_max in hypoxia.

Bearing in mind the limitations of two-species comparisons for inferring adaptation (Garland and Adolph, 1994), it is possible that some of the species differences observed here reflect overall differences between deer mice and white-footed mice, rather than derived changes in the high-altitude population. Indeed, deer mice have the widest altitudinal range of any North American mammal, from around sea level to over 4300 m elevation, and low-altitude populations can be found across much of the continent (Bedford and Hoekstra, 2015; Natarajan et al., 2015; Snyder et al., 1982). However, many previous studies of aerobic performance and cardiorespiratory physiology have found that high-altitude deer mice are distinct from both low-altitude conspecifics and low-altitude white-footed mice, reflecting derived changes in physiology in the high-altitude population (Cheviron et al., 2013; Tate et al., 2020; Ivy and Scott, 2017; Ivy et al., 2020). Adrenergic control of the cardiovascular system is an important determinant of aerobic performance, so increased capacity for adrenergic control may be a key underlying mechanism for the adaptive increase in thermogenic capacity in highlanders (Cheviron et al., 2013; Tate et al., 2020).

## 5 Conclusions

Our study contributes to the emerging evidence that deer mice have adapted to high altitude through evolved changes in several aspects of cardiorespiratory and metabolic physiology that contribute to augmenting aerobic capacity in chronic hypoxia (Lui et al., 2015; Mahalingam et al., 2017; Tate et al., 2017, 2021; Ivy et al., 2020; Wearing et al., 2021; West et al., 2021a,b). In particular, higher maximal cardiac output and tissue O_2_ extraction contribute to augmenting thermogenic *V*O_2_max in chronic hypoxia in highland deer mice compared to their lowland counterparts (Tate et al., 2020). Here, we show that this is associated with increased capacity for regulation of the heart and vasculature by adrenergic receptors. Highlanders had a greater capacity than lowlanders to elevate heart rate via stimulation of β_1_-adrenergic receptors, which may help augment maximal cardiac output. Highlanders also appeared to maintain the capacity for vascular regulation by α-adrenergic receptor stimulation, potentially to preserve the effective redistribution of blood flow to active tissues and augment O_2_ extraction. Therefore, our findings suggest that autonomic regulation of the cardiovascular system has evolved in highland deer mice to help them not only survive but thrive in the challenging environment at high altitude.

## CRediT authorship contribution statement

**Oliver H. Wearing:** Conceptualization, Methodology, Formal analysis, Investigation, Writing – original draft, Writing – review & editing, Visualization. **Derek Nelson:** Methodology, Investigation, Writing – review & editing. **Catherine M. Ivy:** Methodology, Investigation, Writing – review & editing. **Dane A. Crossley:** II, Methodology, Resources, Writing – review & editing, Funding acquisition. **Graham R. Scott:** Conceptualization, Methodology, Resources, Writing – review & editing, Funding acquisition.

## Declaration of competing interest

The authors declare that they have no known competing financial interests or personal relationships that could have appeared to influence the work reported in this paper.
