# Supplementary material for: Adrenergic control of the cardiovascular system in deer mice native to high altitude
Source: Curr Res Physiol. 2022 Jan 29;5:83–92. doi: 10.1016/j.crphys.2022.01.006 (PMC8829085; doi:10.1016/j.crphys.2022.01.006)
Supplement: Multimedia component 1 [file mmc1.docx]

**Adrenergic control of the cardiovascular system in deer mice native to high altitude**

Oliver H. Wearing^*,1^, Derek Nelson^2^, Catherine M. Ivy^1^, Dane A. Crossley II^2^, and Graham R. Scott^1^.

*^1^Department of Biology, McMaster University, Hamilton, ON, Canada; ^2^Department of Biological Sciences, University of North Texas, Denton, TX, USA.*

^*^ Author for correspondence ([wearingo@mcmaster.ca](mailto:wearingo@mcmaster.ca))

**Supplementary Tables**

**Table S1.** Results of statistical comparisons using linear mixed models on body and heart mass data.

| Trait | Animal mass effect | Species (*s*) effect | Acclimation (*a*) effect | *s* x *a* effect |
| --- | --- | --- | --- | --- |
| Body mass^1^ | NA | P = 0.0015* | P = 0.8012 | P = 0.8865 |
|  |  | F_1,23_ = 16.7977 | F_1,23_ = 0.0644 | F_1,23_ = 0.0207 |
| Total ventricle mass | P < 0.0001* | P = 0.9505 | P = 0.4575 | P = 0.9618 |
|  | F_1,23_ = 41.9719 | F_1,23_ = 0.0039 | F_1,23_ = 0.5711 | F_1,23_ = 0.0023 |
| RV mass | P < 0.0001* | P = 0.0878 | P = 0.1495 | P = 0.7476 |
|  | F_1,23_ = 39.0714 | F_1,23_ = 3.1795 | F_1,22_ = 2.2242 | F_1,22_ = 0.1061 |
| LV+S mass | P < 0.0001* | P = 0.4807 | P = 0.1726 | P = 0.8639 |
|  | F_1,23_ = 26.8873 | F_1,23_ = 0.5138 | F_1,22_ = 1.9814 | F_1,22_ = 0.0301 |
| RV/(LV+S) | NA | P = 0.0004* | P = 0.0155* | P = 0.7477 |
|  |  | F_1,24_ = 16.5083 | F_1,24_ = 6.7955 | F_1,24_ = 0.1058 |
| ^1^Family included in model as a significant (P < 0.05) random factor. *Significant effect, P < 0.05. Statistical tests of ventricle mass data used absolute values and accounted for body mass as a covariate, but the data are reported relative to body mass in Table 1. RV, right ventricle; LV+S, left ventricle and septum; RV/LV+S, right ventricle to left ventricle and septum ratio. | | | | |

**Table S2.** Results of statistical comparisons using linear mixed models on the effects of species, hypoxia acclimation and receptor-specific adrenergic drugs on cardiovascular function.

| Trait | Species (*s*) effect | Acclimation (*a*) effect | Drug (*d*) effect | *s* x *a* effect | *s* x *d* effect | *a* x *d* effect | *s* x *a* x *d* effect |
| --- | --- | --- | --- | --- | --- | --- | --- |
| *f*_H_ | P = 0.6697 | P = 0.6702 | P < 0.0001* | P = 0.5498 | P = 0.0301* | P = 0.3263 | P = 0.6400 |
|  | F_1,25_ = 0.1864 | F_1,25_ = 0.1857 | F_2,50_ = 117.0944 | F_1,25_ = 0.3676 | F_2,50_ = 3.7601 | F_2,50_ = 1.1454 | F_2,50_ = 0.4503 |
| *P*_mean_ | P = 0.4594 | P = 0.6383 | P < 0.0001* | P = 0.3620 | P = 0.2369 | P = 0.0286* | P = 0.0753 |
|  | F_1,24_ = 0.5654 | F_1,24_ = 0.2266 | F_2,24_ = 255.6394 | F_1,24_ = 0.8634 | F_2,24_ = 1.5306 | F_2,24_ = 4.1371 | F_2,24_ = 2.8871 |
| *Significant effect, P < 0.05. *f*_H_, heart rate; *P*_mean_, mean arterial pressure. | | | | | | | |

**Table S3.** Results of statistical comparisons using linear mixed models on the effects of species and hypoxia acclimation on the change in cardiovascular variables by adrenergic stimulation.

| Trait | Species (*s*) effect | Acclimation (*a*) effect | *s* x *a* effect |
| --- | --- | --- | --- |
| Δ*f*_H_ by β_1_-adrenergic receptors | P = 0.0234* | P = 0.5173 | P = 0.6005 |
|  | F_1,25_ = 5.8301 | F_1,25_ = 0.4314 | F_1,25_ = 0.2813 |
| Δ*P*_mean_ by ɑ-adrenergic receptors | P = 0.1352 | P = 0.2152 | P = 0.0308* |
|  | F_1,11_ = 2.6000 | F_1,11_ = 1.7294 | F_1,11_ = 6.1287 |
| *Significant effect, P < 0.05. *f*_H_, heart rate; *P*_mean_, mean arterial pressure. | | | |

**Table S4.** Results of statistical comparisons using linear mixed models on the effects of species and hypoxia acclimation on left ventricle parameters measured using intraventricular pressure-volume catheter.

| Trait | Body mass effect | Species (*s*) effect | Acclimation (*a*) effect | *s* x *a* effect |
| --- | --- | --- | --- | --- |
| *f*_H_ | NS | P = 0.2378 | P = 0.0593 | P = 0.1157 |
|  |  | F_1,9_ = 1.5988 | F_1,9_ = 4.6541 | F_1,9_ = 3.0306 |
| Stroke volume | P < 0.0001* | P = 0.0003* | P = 0.0666 | P = 0.2400 |
|  | F_1,8_ = 63.8379 | F_1,8_ = 35.4430 | F_1,8_ = 4.5027 | F_1,8_ = 1.6114 |
| Cardiac output | P = 0.0004* | P = 0.0100* | P = 0.4752 | P = 0.0079* |
|  | F_1,8_ = 34.5960 | F_1,8_ = 11.2374 | F_1,8_ = 0.5612 | F_1,8_ = 12.3722 |
| Stroke work | P = 0.0002* | P = 0.0011* | P = 0.0233* | P = 0.3779 |
|  | F_1,8_ = 41.3624 | F_1,8_ = 25.0122 | F_1,8_ = 7.8259 | F_1,8_ = 0.8713 |
| *V*_max_ | P < 0.0001* | P = 0.0043* | P = 0.7546 | P = 0.9717 |
|  | F_1,8_ = 76.2661 | F_1,8_ = 15.4679 | F_1,8_ = 0.1047 | F_1,8_ = 0.0013 |
| *V*_min_ | P = 0.0018 | P = 0.0546 | P = 0.2122 | P = 0.7929 |
|  | F_1,8_ = 20.9681 | F_1,8_ = 5.0597 | F_1,8_ = 1.8379 | F_1,8_ = 0.0737 |
| *P*_max_ | NS | P = 0.3541 | P = 0.1007 | P = 0.2018 |
|  |  | F_1,9_ = 0.9543 | F_1,9_ = 3.3442 | F_1,9_ = 1.8958 |
| *P*_min_ | NS | P = 0.6659 | P = 0.0409* | P = 0.6763 |
|  |  | F_1,9_ = 0.1993 | F_1,9_ = 5.6904 | F_1,9_ = 0.1862 |
| *P*_mean_ | NS | P = 0.4502 | P = 0.5919 | P = 0.1982 |
|  |  | F_1,9_ = 0.6231 | F_1,9_ = 0.3089 | F_1,9_ = 1.9295 |
| *P*_dev_ | NS | P = 0.2929 | P = 0.0456* | P = 0.1573 |
|  |  | F_1,9_ = 1.2479 | F_1,9_ = 5.3756 | F_1,9_ = 2.3801 |
| ^1^EF | NS | P = 0.1764 | P = 0.0094* | P = 0.0035* |
|  |  | F_1,7_ = 2.7737 | F_1,7_ = 15.4298 | F_1,7_ = 24.1286 |
| d*P*/dt_max_ | NS | P = 0.4657 | P = 0.2354 | P = 0.8387 |
|  |  | F_1,9_ = 0.5803 | F_1,9_ = 1.6172 | F_1,9_ = 0.0439 |
| E_es_ | NS | P = 0.0665 | P = 0.9468 | P = 0.4243 |
|  |  | F_1,9_ = 4.3571 | F_1,9_ = 0.0047 | F_1,9_ = 0.7005 |
| ^1^Sex and family included in model as significant (P < 0.05) random factors. *Significant effect, P < 0.05. Statistical tests of all volume data used absolute values and accounted for body mass as a covariate, but the data are reported relative to body mass in Table 2. NS denotes situations in which body mass was omitted as a factor in final linear models because initial tests suggested that its effect did not near significance (P ≥ 0.1). *f*_H_, heart rate; *V*_max_, maximum left ventricle volume; *V*_min_, minimum left ventricle volume; *P*_max_, maximum left ventricle pressure; *P*_min_, minimum left ventricle pressure; *P*_mean_, mean left ventricle pressure; *P*_dev_, pressure developed by left ventricle contraction; EF, ejection fraction; d*P*/dt_max_, maximum derivative of pressure; E_es_, end-systolic elastance, which is the slope of the end-systolic pressure-volume relationship. | | | | |
